# Gabriel Richet: when medicine combines with courage

**DOI:** 10.1186/s12882-018-0887-4

**Published:** 2018-04-19

**Authors:** Pierre Ronco

**Affiliations:** 1 Inserm UMR_S 1155, Hôpital Tenon, 4 rue de la Chine, 75020 Paris, France; 2 Sorbonne Universités, UPMC Univ Paris 06, 75005 Paris, France; 3 AP-HP, Hôpital Tenon, Service de Néphrologie et Dialyses, Paris, France

## Abstract

Gabriel Richet, one of the fathers of the French and international Nephrology, was a man remarkable for his courage, vision and empathy. He was proud and brave, and he presented himself proud of being brave. He opens his interview speaking about his youth, when he was injured, and went back to the fight. He cites the number of stitches he received, but doesn’t cite being decorated with the Légion d’Honneur, one of the highest honours of the French Republic. This anecdote perfectly illustrates his elegance and detachment from awards and self-satisfaction. Gabriel Richet was a visionary. He was the first one to use the artificial kidney in France. Together with Jean Hamburger and Jean Crosnier at the Necker hospital, he developed the concept of renal intensive care and, later on, he was one of the first to develop the concept of translational nephrology.

At a time when medical writing was not acknowledged, he authored almost 400 manuscripts indexed on Medline. He was over 90 when his last papers, dealing with the history of Nephrology, were published, some of them as sole author.

In the interview, as well as in his life, he did not renounce to a provocative self-irony. A physician should never give up, he should assume the full responsibility of his actions, and practice medicine with the heart: “I am like the Queen of Holland, whose motto is: I will maintain”. In our uncertain, unsafe, fragile and turbulent world, there is no better motto for us all.

## Editorial

As we are opening this beautiful series devoted to the Pioneers of Nephrology, I could not help but ponder Richet’s legacy. Gabriel Richet was my Mentor, and the Mentor of so many others.

I could start by saying without reservation that Gabriel Richet was one of the giants of French and international nephrology. However, his stature cannot only be measured by the impressive list of honours and achievements that marked his long career [1–3]. Actually, this inescapable enumeration would fail to portray the great man and Mentor he was.

It is not by chance that his self-presentation in this interview starts with “I am a man who always had a good luck. I have done many silly things and overall their results have been quite good. That’s what it’s all about. I was very bold during the war in 1939–1940, and in 1944–45, and in the time between (...). I was injured, a bullet entered the top part of my right thigh and stopped by the left femoral artery, 60 cm of stitches and 60 days later, I was back to the front line. That is to say that I have a rather adventurous spirit.” [4].

Gabriel Richet was a man remarkable for his courage, his vision, and his empathy.

He was proud and brave, and proud of being brave, as he presented himself.

The story tells that, at 23 years of age, soon after having passed the internship exam, he was enrolled in the army and took part in the France military campaign. After that he returned to medical activities, while actively participating to the French Resistance with all his family members, most of them being imprisoned or deported. Immediately after the Liberation of Paris, in 1944, he left again for the army under the high command of General Leclerc, who freed Strasbourg in November 1944. In the first months of 1945, fights continued near Colmar in Alsace; there, Gabriel Richet was active as a doctor of the French Commandos.

He was injured, and went back to the fight, received three military citations, and was decorated with the award of Chevalier de la Légion d’Honneur by Général de Gaulle in April 1945 [1, 2]. That he cites the number of stitches, but doesn’t cite the Légion d’Honneur, one of the highest honours of the French Republic, perfectly illustrates the elegance and detachment from awards and self-satisfaction.

Later in his interview, he points out how war taught him to choose and be focused: An older lieutenant in the Commando told me: “If you have two goals, you’ll reach neither of them and you have a high risk of being killed. No second goal”. Every year until the age of 97, Gabriel Richet had dinner with the vanishing members of his Commando. One survivor attended his funeral as a testimony to the importance of this period of his life, which he recalled very often in private.

Gabriel Richet was a visionary. He was the first one to use the artificial kidney in France. Together with Jean Hamburger and Jean Crosnier at the Necker hospital, he developed the concept of renal intensive care aimed at correcting disorders of the “milieu intérieur”, thereby markedly improving the prognosis of acute kidney injury.

He worked at a time when medical writing was not fully acknowledged, yet, he authored almost 400 scientific papers indexed on Medline; browsing the titles now gives an idea of his profound curiosity and his capacity to discuss the “common thoughts”. Even more remarkably, he never gave up: he was over 90 when his last papers, dealing with the history of Nephrology, were published, some of them as sole author [5–12]. It is almost impossible to describe here the works and discoveries that took place at the Necker then at the Tenon Hospital. Suffice to say that Gabriel Richet had his own group of research and was proud of the discovery of the dark cells of the collecting duct, now called intercalated cells, which play an important role in acid-base control.

Never in his life, Gabriel Richet looked for easy ways, or for public recognition, which he elegantly accepted. In 1961 he left the Mecca of the newborn discipline of Nephrology, Necker Hospital, to move to an old hospital, Tenon, in a peripheral area of Paris, which no one would call “chic”.

In Tenon, Gabriel Richet not only developed an international center of Nephrology, he created a unique atmosphere of intellectual curiosity and creativity by providing support and guidance to each member of the team, while at the same time leaving the freedom for all to develop their own projects.

Thanks to his generosity and his warm personality, most of those who worked with him would consider him a second father, a position that he accepted and filled with humour and joviality.

Three years after his death, when his former fellows meet, their first words are often for Richet, recalling the exciting time we all had at the Tenon Hospital under his leadership.

His interview ends with a motto: “Je maintiendrai”. Throughout his long interview, Gabriel Richet did not renounce to a touch of provocation, slightly smoothed by self-irony: “I am like the Queen of Holland, whose motto is: I will maintain”.

A physician should never give up; he should assume the full responsibility of his actions, and practice medicine with the heart.

In our uncertain, unsafe, fragile and turbulent world, there is no better motto for us all.
